# The resting frequency of echolocation signals changes with body temperature in the hipposiderid bat *Hipposideros armiger*

**DOI:** 10.1242/jeb.243569

**Published:** 2022-02-03

**Authors:** Diana Schoeppler, Annette Denzinger, Hans-Ulrich Schnitzler

**Affiliations:** Animal Physiology, Institute for Neurobiology, Faculty of Science, University of Tübingen, 72076 Tübingen, Germany

**Keywords:** Audio–vocal feedback control, Auditory fovea, CF-FM echolocation signals, Doppler shift compensation, Flutter detection

## Abstract

Doppler shift (DS) compensating bats adjust in flight the second harmonic of the constant-frequency component (CF_2_) of their echolocation signals so that the frequency of the Doppler-shifted echoes returning from ahead is kept constant with high precision (0.1–0.2%) at the so-called reference frequency (*f*_ref_). This feedback adjustment is mediated by an audio–vocal control system that correlates with a maximal activation of the foveal resonance area in the cochlea. Stationary bats adjust the average CF_2_ with similar precision at the resting frequency (*f*_rest_), which is slightly below the *f*_ref_. Over a range of time periods (from minutes up to years), variations of the coupled *f*_ref_ and *f*_rest_ have been observed, and were attributed to age, social influences and behavioural situations in rhinolophids and hipposiderids, and to body temperature effects and flight activity in *Pteronotus parnellii*. We assume that, for all DS-compensating bats, a change in body temperature has a strong effect on the activation state of the foveal resonance area in the cochlea, which leads to a concomitant change in emission frequency. We tested our hypothesis in a hipposiderid bat, *Hipposideros armiger*, and measured how the circadian variation of body temperature at activation phases affected *f*_rest_. With a miniature temperature logger, we recorded the skin temperature on the back of the bats simultaneously with echolocation signals produced. During warm-up from torpor, strong temperature increases were accompanied by an increase in *f*_rest_, of up to 1.44 kHz. We discuss the implications of our results for the organization and function of the audio–vocal control systems of all DS-compensating bats.

## Abstract (precis)

**Summary:** In *Hipposideros armiger*, the variation of body temperature during activation phases results in a concomitant change in emission frequency.

## INTRODUCTION

The auditory system of bats using the flutter detection echolocation strategy is highly specialized for the extraction of behaviourally relevant information in echoes from fluttering prey (for reviews, see Neuweiler et al., 1980; Schnitzler and Ostwald, 1983; Schnitzler and Denzinger, 2011; Fenton et al., 2012; Denzinger et al., 2018). Flutter-detecting foragers emit echolocation signals consisting of a long constant-frequency component, followed by a short downward frequency-modulated terminal part at high duty cycle, with the highest amplitude in the second harmonic (CF_2_). In flight, these bats use an extremely precise audio–vocal feedback control system to adjust the emission frequency so that the CF_2_ from echoes, returning from stationary targets ahead, is kept constant at the so-called reference frequency (*f*_ref_), which is defined as average CF_2_ from all echoes (Schnitzler, 1968, 1973). By lowering the emission frequency, bats compensate for Doppler shifts (DS) in echoes from targets ahead over the whole range of their flight speeds (Schnitzler and Henson, 1980; Schoeppler et al., 2018). This echolocation behaviour has therefore been termed DS compensation (Schnitzler, 1967), and is found in rhinolophid (Schnitzler, 1968) and hipposiderid bats (Gustafson and Schnitzler, 1979) and in the mormoopid bat *Pteronotus parnellii* (Schnitzler, 1970). In playback experiments, stationary horseshoe bats also compensated for simulated positive DS and adjusted CF_2_ of the playback echoes at *f*_ref_ (Schuller et al., 1974), although negative shifts were not compensated for.

DS-compensating bats possess an auditory fovea in the cochlea, consisting of a morphologically specialized resonance area with a disproportionally high overrepresentation of hair cells tuned to frequencies at or near *f*_ref_ (Suga et al., 1975; Bruns, 1976; Schuller and Pollak, 1979; Kössl and Vater, 1985; for reviews, see Kössl and Vater, 1995; Vater, 2004). By the adjustment of echo CF_2_ at *f*_ref_, DS compensation leads to a maximal activation of the resonance system in the inner ear. Afferent connections from the auditory fovea in the cochlea project to foveal areas in higher centres of the entire ascending auditory pathway with an overrepresentation of many sharply tuned neurons with best frequencies at or near *f*_ref_ (reviewed in Neuweiler et al., 1980; Suga, 1984; Schnitzler and Denzinger, 2011). The auditory fovea is also featured in the auditory threshold, where the threshold minimum is narrowly tuned to the *f*_ref_ with a distinct threshold increase to lower frequencies (reviewed in Schnitzler and Denzinger, 2011). The sensory information encoded in these foveal areas acts on motor areas for vocal control and determines the CF_2_ of emitted echolocation signals, thus completing the audio–vocal control loop (Schuller and Rübsamen, 1981; Rübsamen and Betz, 1986; Metzner, 1989; Kössl and Vater, 2000).

When flutter-detecting bats vocalize while resting, they keep the CF_2_ of their signals similarly constant with high precision at the so-called resting frequency (*f*_rest_; CF_2_ average of emitted pulses) with deviations of not more than 0.1–0.2% (reviewed in Schnitzler and Denzinger, 2011; Schoeppler et al., 2018). In all flutter-detecting foragers, *f*_ref_ and *f*_rest_ are tightly coupled with a species-specific offset between the two of not more than a few hundred Hz (reviewed in Schnitzler and Denzinger, 2011). This constancy of *f*_rest_ and *f*_ref_ has been supported in experiments over only very short time periods (i.e. a few seconds). At longer time periods, however, intra-individual variations of *f*_rest_ have been documented for several species of flutter-detecting foragers (rhinolophids: Long and Schnitzler, 1975; Hiryu et al., 2008; Furusawa et al., 2012; hipposiderids: Riquimaroux, 2000; Hiryu et al., 2006; Schoeppler et al., 2018; *P. parnellii*: Suga et al., 1987; Gaioni et al., 1990). A change of *f*_rest_ over the course of several days or months often occurred as a gradual decrease with time, sometimes accompanied by reversion back to the original *f*_rest_. The highest individual variations have been observed in hipposiderids, in a long-term study of *Hipposideros terasensis*, all bats decreased or increased their *f*_rest_ in the same direction with variations of 3 kHz on average (Hiryu et al., 2006). Integration of new individuals to a preexisting colony usually provoked an increase in frequency and isolations and always resulted in large drops in *f*_rest_. A common feature of all these reports is that the documented intra-individual frequency shifts relate to specific behavioural situations and/or to a specific social context (Hiryu et al., 2006) but do not offer a physiological mechanism which explains them.

Physiological mechanisms that may be responsible for variations of *f*_rest_ and *f*_ref_ have only been studied in the mormoopid bat *P. p. parnellii* (Henson et al., 1990; Huffman and Henson, 1991, 1993a,b). These studies found that changes of the cochlear resonance frequency (CRF), which were induced by flight activity, body temperature and contralateral noise, resulted in variations of the *f*_rest_ and the coupled *f*_ref_ in bats sitting on a swinging pendulum (Huffman and Henson, 1993a). These changes also had an effect on the best frequencies of neurons in the cochlear nucleus and in the inferior colliculus (Huffman and Henson, 1991, 1993b). The measured effects of body temperature reported in these experiments were somewhat contrasting. In the first study (Henson et al., 1990), the CF_2_ changes did not consistently correlate with temperature changes induced by flight activity. However, in the following experiments (Huffman and Henson, 1991, 1993a,b), a clear temperature dependency was demonstrated when the animals were warmed up by a heat lamp.

These studies on the mormoopid bat suggest that *f*_rest_ and *f*_ref_ might be labile and dependent on the physiological state of a bat. Results suggest that the temperature-induced frequency shifts result from changes of the resonance properties of the foveal area in the cochlea. The closely related rhinolophid and hipposiderid bats also compensate for DS, but they are phylogenetically more distant from *P. parnellii* (Jones and Teeling, 2006). Their auditory fovea is also based on specific resonance properties of the cochlea but differs in its morphology and physiology from that of *P. parnellii* (Henson et al., 1985; Kössl, 1994; for reviews, see Vater, 2004; Neuweiler, 2003). Hence, mechanisms which induce changes in resonance properties in *P. parnellii* are not necessarily those that cause the changes in rhinolophids and hipposiderids.

The aim of this study is to understand how a physiological parameter (body temperature) affects *f*_rest_ of the echolocation signals in the hipposiderid bat *Hipposideros armiger*. We hypothesized that the activation of the foveal resonance system in the cochlea of this species (and presumably of other rhinolophid and hipposiderid bats) will change with body temperature and should lead to a concomitant frequency change of the emitted echolocation signals. With an external miniature temperature logger placed between the scapulae, we measured the diurnal change of skin temperature continuously over periods of 34 h, recorded simultaneously the echolocation signals of vocalizing bats, determined their resting frequency, and correlated *f*_rest_ with the measured skin temperature. We discuss our results with regard to our current view on the organization of the audio–vocal control system for DS compensation, and in view of the resistance of DS compensation to disturbances by temperature effects.

**List of symbols and abbreviations**

CF_2_: constant-frequency component of the second harmonic
CRF: cochlear resonance frequency
DS: Doppler shift
*f*_max_
: frequency maximum
*f*_ref_
: reference frequency
*f*_rest_
: resting frequency
*t*_max_
: relative skin temperature maximum

## MATERIALS AND METHODS

### Animals and ethical statements

Experiments were conducted with two adult female Vietnamese round-leaf nosed bats, *Hipposideros armiger* (Hodgson 1835), captured in 2009 in the Ba Be National Park in Vietnam with permission from 13 May 2009 granted to the Vietnamese Institute of Ecology and Biological Resources, Hanoi (no. 129/STTNSV). Bats were kept at the animal facility of the Institute for Neurobiology of the University of Tübingen in a room (6×3.6×3 m) under controlled abiotic conditions (12 h:12 h light:dark cycle, 26.6±2°C and 60±5% humidity) and housed separately in two aviaries (2.4×1.2×2 m and 3.2×1.25×2 m). During the non-experimental time, the aviaries were open so that the bats could freely fly in the room. Water and mealworms (larvae of *Tenebrio molitor*) were available *ad libitum* and semi-monthly vitamin and mineral supplements (Nutri-Cal^®^ Albrecht GmbH, Germany; Efaderm^®^ aristavet GmbH & Co., Germany; Korvimin^®^ WDT eG, Germany) were administered. Additionally, crickets (*Gryllus* spp., *Acheta domestica*), locusts (*Schistocerca gregaria*) and beetles (*Zophobas morio*, *Pachnoda marginata*) were hand-fed to the bats.

During this time, bats were trained to carry a miniature temperature logger on their back. Using positive reinforcement with favoured food such as beetles and locusts, the bats learned to climb on the hand of the experimenter. When bats were accustomed to handling, we shaved off a small patch of fur between the shoulder blades and fixed a dummy with water-soluble glue (Mastix watersoluble, GRIMAS^®^ B.V., Holland; residue-free removable, safe for use with children) onto the shaved spot between the shoulder blades to get the bat accustomed to an object. After this habituation procedure, we attached the miniature temperature logger for registration of skin temperature. The mass of the logger was 1.66 g and corresponded to less than 3% of subject body mass, which was 60 g on average. The logger did not affect behaviour. After each session, the logger was removed with water.

The experiments were conducted in accordance with the German Animal Welfare Legislation and did not require explicit approval (letter from the approval authority from 29 March 2012). The license to keep *H. armiger* was issued by the responsible agency (Regierungspräsidium Tübingen, Germany).

### Experimental setup and recordings

During each experiment we recorded the skin temperature between the shoulder blades every minute with an i-Button ETL1 miniature temperature logger (Maxim Integrated, USA). The logger had a temperature range from +15 to +46°C and a resolution of 0.125°C. During this time, we also made continuous sound recordings (PCtape, Animal Physiology, University of Tübingen). The echolocation signals were picked up with a custom-made ultrasonic microphone (frequency response within 5 dB in the range of the echolocation signals), amplified, digitized with a sampling rate of 480 kHz, 16-bit, and stored as .wav files. The microphone was positioned around 80 cm under the bat's preferred resting place and recorded all emitted signals with sufficient quality independent of the bat's position in the aviary.

Each bat was tested in two separate sessions, each lasting 34 h. The recordings started approximately 5 h before the lights were turned off. The experimenter entered the room 1–2 h after the beginning of this dark phase and stayed in the room for up to 25 min conducting usual husbandry activities. Additionally, the experimenter entered the room again at the end of the dark phase and checked visually the condition of the animal and the fit of the logger. During the experiment phase, bats had been familiar both with this procedure and the experimenter for several months prior. After warming up, bats generally flew to the experimenter and landed nearby or even on the hand to receive crickets or beetles.

### Data analysis and statistics

**Fig. 1.**
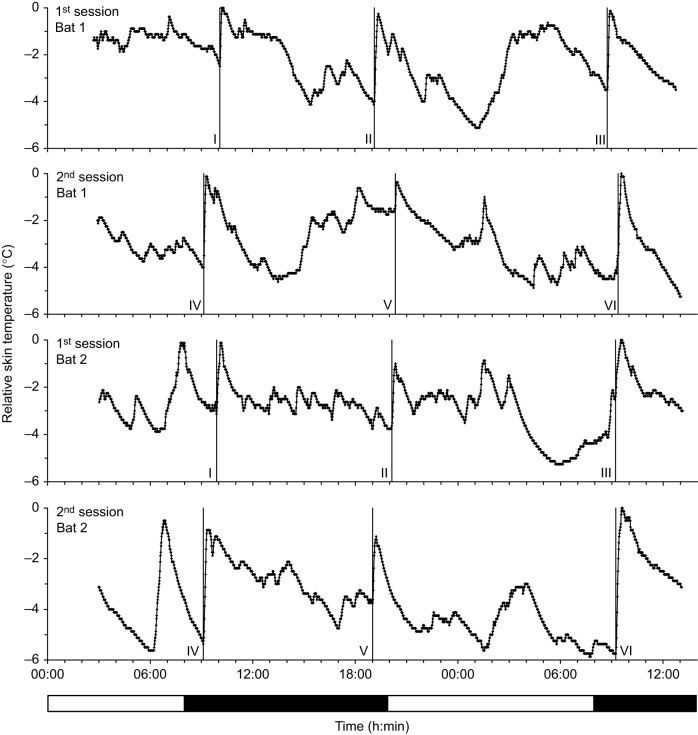
**Relative skin temperature of bat 1 and bat 2.** The temperature was measured every minute over a period of 34 h in two sessions per bat. Skin temperature is shown relative to the maximum during each period. Bats increased body temperature when the experimenter entered the room which is marked by a vertical line (no. I–VI). The bar below indicates the light:dark cycle (12 h:12 h). The light was turned off at 08:00 h and turned on at 20:00 h.

**Fig. 2.**
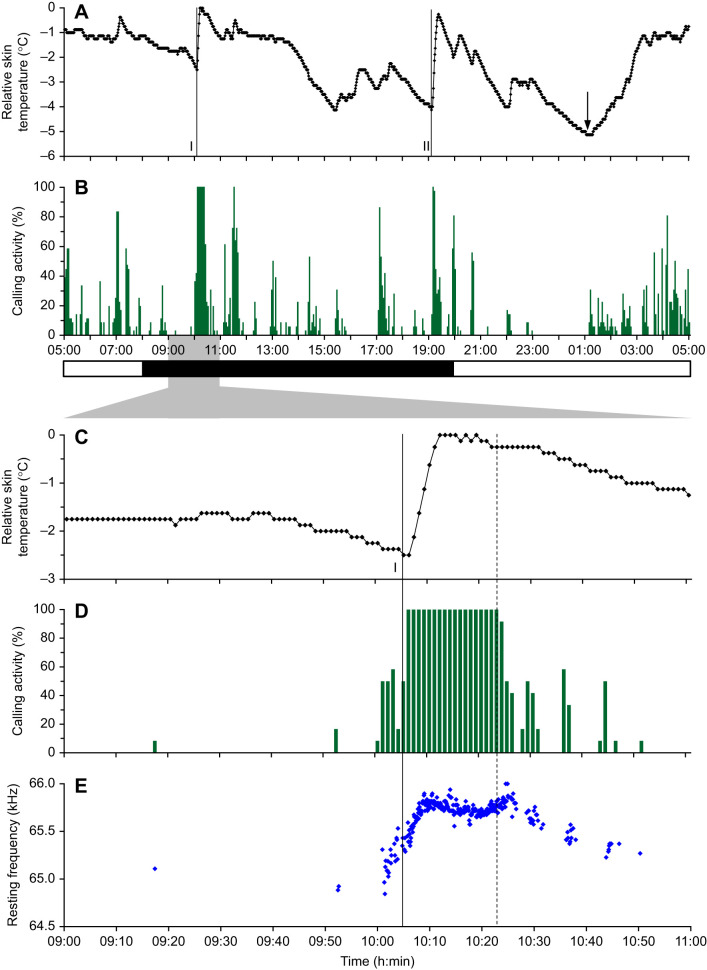
**Echolocation signal emission and skin temperature course over 24 h and one activation in detail.** Relative skin temperature (A,C), calling activity (B,D) and resting frequency (E) of one bat during a 24 h period (A,B) and the 2 h period (C–E) around the first activation (I) by the experimenter (black line). Skin temperature is shown relative to the daily maximum (32.1°C). The minimum is marked with an arrow (A) and occurred at the end of the longest silent period. Calling activity was depicted in time bins of 3 min (B) and 1 min (D). Each frequency value in E represents the resting frequency in a 5 s slot. The vertical lines in C–E mark the instant of time when the experimenter entered (solid line) and left (dotted line) the husbandry room.

**Fig. 3.**
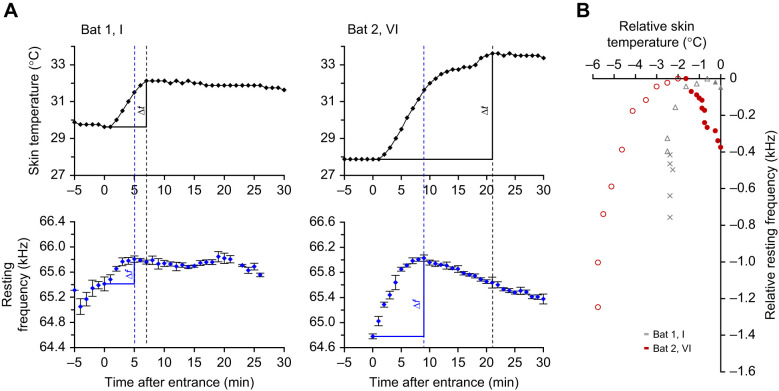
**Exemplary course of skin temperature and resting frequency during activation, with and without calling activity before.** (A) Absolute skin temperature and resting frequency in the time periods 5 min before and 30 min after activation by the experimenter (no. I of bat 1 and no. IV of bat 2; see Fig. S1). (B) Correlation between the same data normalized to their maximum values. Each dot corresponds to a 1 min period, the resting frequency was averaged (mean±s.d.). In A, the dotted blue lines indicate the frequency maxima and the dotted black lines the skin temperature maxima. Solid lines mark the temperature and frequency rise from activation to the maximum. In B, × symbols depict values 5 min before activation, open symbols depict the values from activation until the frequency maximum is reached and filled symbols depict the values to the temperature maximum after the frequency maximum was reached.

**Fig. 4.**
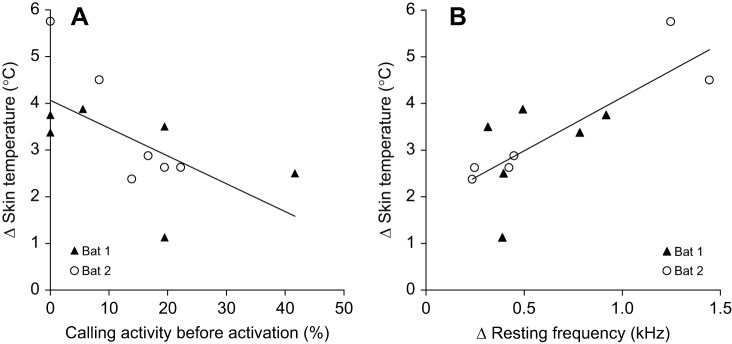
**Relationship between skin temperature and echolocation signal emission.** (A) Correlation between the increase in skin temperature and calling activity before activation and (B) the increase in resting frequency and increase in skin temperature for all six activations in the two bats with regression lines. The calling activity before activation (A) is based on the time span 3 min before the experimenter entered the room. (A) Spearman's ρ=−0.675, *N*=12, *P*=0.0160; (B) Spearman's ρ=0.79, *N*=12, *P*=0.0022.

**Fig. 5.**
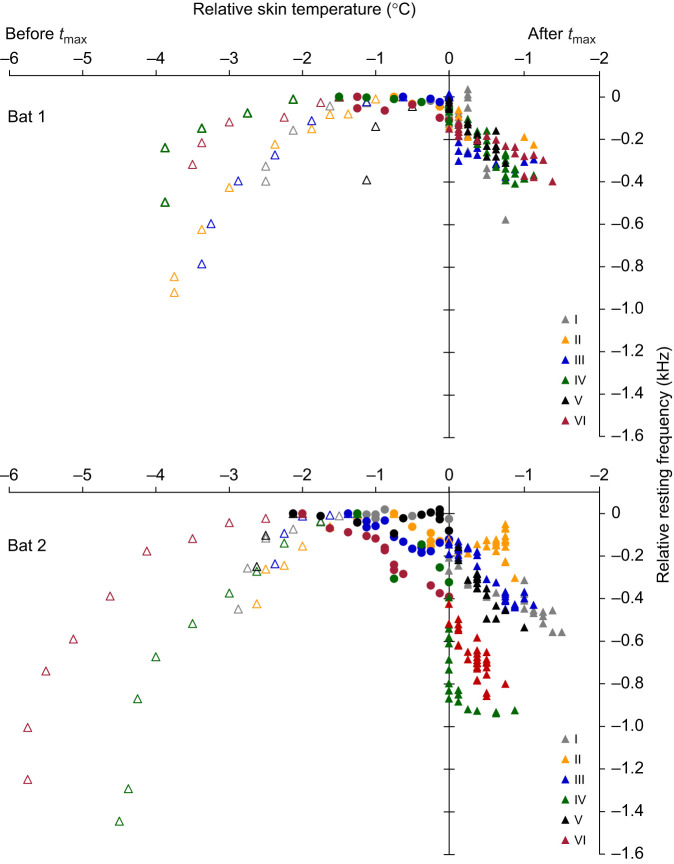
**Correlation between relative resting frequency and the relative skin temperature of bat 1 and bat 2 for all activations (I–VI).** Each curve presents data from activation to the frequency peak (open symbols), between frequency and temperature peak (filled circles), and data after the temperature peak (filled triangles). The latter is given on a second negative *x*-axis to the right for better separation of data points. Each dot corresponds to the average value of a 1 min period. Per bat (*N*=6) sign test *P*=0.031.

Data from the temperature logger were exported via an USB reader and OneWireViewer software (both Maxim Integrated). Depending on the fit of the logger, the maximum skin temperature varied up to 3°C between the sessions. Therefore, we calculated the skin temperature relative to the maximum of one session (Figs 1 and 2) and relative to the maximum of each rise (Figs 3B, 4 and 5; Fig. S1). Temperature values were not normally distributed (Shapiro–Wilk test, *P*<0.05). Therefore, a Wilcoxon rank sum test was conducted, to test for differences in skin temperature between light and dark phases with two complete 24 h cycles lasting from 08:00 to 07:59 h per bat.

Sound analysis was conducted with the software Selena (University of Tübingen) in colour spectrograms. We first measured the calling activity of a bat over 24 h to test whether it was associated with change in skin temperature. To determine calling activity, we subdivided the 24 h recording in time bins of 3 min. For every time bin, we determined in steps of 5 s whether the bat was vocalizing. We defined calling activity if the bat emitted at least 10 signals within the 5 s time slot. The calling activity within the 3 min time bin was calculated as the percentage of calling activity from the thirty-six 5 s time slots of each 3 min bin. For example, the calling activity in the 3 min time bin was 100% if the bat emitted at least 10 calls during each 5 s interval. Because a normal distribution was not observed across the data (Shapiro–Wilk test, *P*<0.05), we performed a Wilcoxon rank sum test to test whether the calling activity differed between the light and dark phase.

To measure changes in CF_2_ caused by the activation of the bats, we determined the CF_2_ of resting signals, also known as resting frequency (*f*_rest_), emitted 2 h before and 2 h after the entrance of the experimenter. Calls were displayed in a 5 s window as spectrograms between 60 and 75 kHz using a fast Fourier transform with 8192 points (zero padding), which resulted in a frequency resolution of 20.25 Hz. *f*_rest_ was manually measured as average maximum frequency over all calls in the 5 s window. We only used 5 s time slots with at least 10 signals as single calls, and in some cases the first few calls (up to three) in slots with calling activity were very different in frequency compared with the others. They were excluded from *f*_rest_ measurements.

Further, we correlated the increase in skin temperature from beginning of activation to its maximum with the percentage calling activity in the 3 min time span before the entrance of the experimenter, and tested it using a Spearman correlation. Although the Shapiro–Wilk test (*P*>0.05) indicated a normal distribution, we conducted a non-parametric test to reduce the impact of outliers. To correlate the increase in skin temperature with the increase of *f*_rest_ from activation to its maximum, we averaged the *f*_rest_ per minute. We tested for significance with a Spearman correlation (normal distribution was not observed, Shapiro–Wilk test, *P*<0.05).

We plotted the normalized *f*_rest_ and the normalized skin temperature over time from the point of entrance to the respective maximum. Additionally, we determined the minimum in skin temperature from the peak in skin temperature within 30 min thereafter. We fitted the correlation with linear trend lines to the increasing part after activation until frequency maximum, and to the decreasing part from skin temperature maximum until its described minimum. The slopes of these trend lines were statistically tested with the sign test (Sokal and Rohlf, 1995).

## RESULTS

### Diurnal rhythm of skin temperature

In two individuals of *H. armiger* (referred to hereafter as ‘bat 1’ and ‘bat 2’), skin temperature was continuously measured every minute in two sessions, each lasting 34 h. Skin temperature was never stable. However, the diurnal pattern of the change in skin temperature was rather similar between both session and animal, and indicated the influence of the light:dark cycle as well as the influence of external stimuli, e.g. the entrance of the experimenter (Fig. 1). Skin temperature was slightly higher during the dark phase, when the nocturnal bats were more active than during the light phase. Relative to the normalized maximum skin temperature (*t*_max_) reached in a given session, the median of the skin temperature was 2.1°C (bat 1) and 2.8°C (bat 2) below *t*_max_ in the dark phase and 2.9°C (bat 1) and 4.3°C (bat 2) below *t*_max_ in the light phase (bat 1: *Z*=−10.35, *n*_light_=1440, *n*_dark_=1440, *P*<0.0001; bat 2: *Z*=−23.26, *n*_light_=1440, *n*_dark_=1440, *P*<0.0001). The highest difference to *t*_max_ was measured during the light phase with relative values of 4.9 to 5.9°C below *t*_max_. The strongest effects of distinct skin temperature rises were observed when the experimenter entered the room. After this external stimulus, the *t*_max_ was reached within 4–21 min (Table 1) and deceased afterwards at a slower rate. The relative increase in skin temperature depended on the skin temperature prior to stimulation and was higher at initially low skin temperatures (Fig. 1). For further analysis of temperature effects on *f*_rest_, we concentrated only on the distinct skin temperature increases resulting from the activation of the bats by the experimenter (see Fig. 1).

**Table 1.**
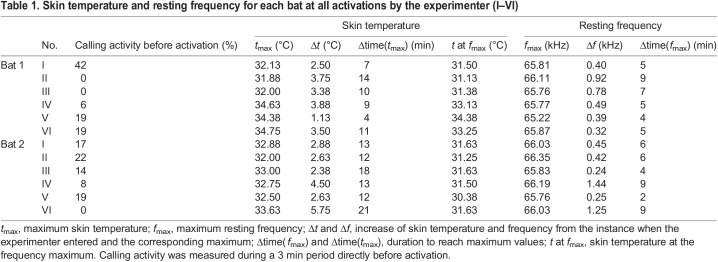
Skin temperature and resting frequency for each bat at all activations by the experimenter (I–VI)

### Variation of skin temperature and calling activity

The diurnal calling activity pattern is characterized by periods lasting up to 26 min where bats emitted more or less continuous calls separated by longer silent periods (Fig. 2A). Calling activity was influenced by the light:dark cycle as well as by external stimuli (Fig. 2). Overall, average calling activity during light periods (average 9%) did not significantly differ from calling activity during dark periods (average 12%; *Z*=−0.3, *n*_light_=241, *n*_dark_=242, *P*=0.7637). Calling activity was high at rising skin temperatures (Fig. 2B,D). The minimum skin temperature occurred during light periods, and in the example in Fig. 2B it was correlated with the lowest calling activity (silence for 2 h). The strongest effects, with a distinct increase in skin temperature and in calling activity, were observed when the experimenter entered the room (Fig. 2; Fig. S1). Therefore, we used this reaction to investigate the relationship between *f*_rest_ and skin temperature. The bat displayed in Fig. 2C,D increased calling activity approximately 4 min before the experimenter entered the room, followed by an increase in skin temperature. Calling activity remained at 100% as long as the experimenter was in the room and dropped rapidly after experimenter exit, accompanied by a slight decrease in skin temperature (Fig. 2C,D).

### Variation of skin temperature and resting frequency

When activated by the experimenter, *H. armiger* reacted not only with an increase in skin temperature and calling activity, but also shifted the CF_2_ of the resting signals (*f*_rest_) continuously towards higher frequencies (Figs 2E and 3A; Fig. S1). Both skin temperature and *f*_rest_ increased steeply to a maximum (Figs 2E and 3A, Table 1; Fig. S1). Generally, bats with a high calling activity in the 3 min prior to activation also had a higher initial skin temperature and a higher initial *f*_rest_, and therefore a smaller increase in each (Table 1, Figs 3A and 4A). Increase in skin temperature and calling activity were inversely correlated (Spearman's ρ =−0.675, *N*=12, *P*=0.0160) (Fig. 4A). The maximum in *f*_rest_ (*f*_max_) was reached earlier (within 2–9 min) than the *t*_max_ (within 4–21 min), except in one case, when the frequency and the skin temperature simultaneously reached the maximum after 4 min (Table 1). The relative increase of the *f*_rest_ ranged between 0.24 and 1.44 kHz with increase rates of 60–160 Hz min^−1^ or 90–350 Hz °C^−1^ (skin temperature). The *t*_max_ was reached on average 6±4 min after the *f*_max_, but the longest interval measured 14 min. The relative increase of skin temperature ranged between 1.13 and 5.75°C, with increase rates between 0.13 and 0.43°C min^−1^. The relative increases in frequency were positively correlated with relative increases in skin temperature (Spearman's ρ=0.79, *N*=12, *P*=0.0022) (Fig. 4B).

In the time span between *f*_max_ and *t*_max_, the skin temperature increased by 1.1°C on average (0.6–2.1°C) in 11 of 12 cases, whereas the *f*_rest_ was decreasing slightly, by an average of 70 Hz. Only in activations IV and VI of bat 2 (Figs 3B and 5) was the *f*_rest_ reduction much higher (values of 320 and 390 Hz, respectively; Table 1, Figs 3 and 5). After the *t*_max_ was reached, skin temperature and *f*_rest_ decreased slowly in both bats (Fig. 5; Fig. S1). Skin temperature dropped at an average rate of 0.04°C min^−1^ and reached a minimum within 18–30 min (0.75–1.5°C below the maximum). *f*_rest_ decreased to the range of 90–600 Hz, with an average rate of 10 Hz min^−1^ or 390±240 Hz °C^−1^ (skin temperature), and 310±160 Hz °C^−1^ (skin temperature) after removal of two bat 2 outliers (IV and VI). Although the peaks of these two variables did not occur at the same time, the *f_r_*_est_ always increased with increasing skin temperature until *f*_max_ was reached (sign test, *P*=0.031 for each bat). Likewise, the *f*_rest_ decreased concurrently with decreasing skin temperature after the *t*_max_ was reached (sign test, *P*=0.031 for each bat) (Fig. 5).

## DISCUSSION

In this study, we investigated the influence of the circadian changes of body temperature on the CF_2_ of resting signals (resting frequency or *f*_rest_) in *H. armiger.* With this approach, we tested the hypothesis that body temperature influences the resonance conditions in the foveal area of the cochlea in a DS-compensating hipposiderid bat. Previous work had suggested this occurs in a mormoopid bat (Huffman and Henson, 1991, 1993a,b), but this had never been tested in hipposiderid or rhinolophid bats before. The concomitant change in cochlear output should lead to a different activation of the audio–vocal control system, which should result in a readjustment of the emission frequency and its rise with increasing body temperature.

The circadian variation of the skin temperature of *H. armiger* was strongly determined by external Zeitgeber (Fig. 1). At the beginning and end of a dark phase, bats reacted to the entrance of the experimenter with a distinct increase of body temperature to a maximal value, which was always accompanied by a continuous emission of signals with rising *f*_rest_ (Fig. 2; Fig. S1). Sometimes bats increased their calling activity prior to experimenter entrance, which may indicate that they were accustomed to the regular husbandry procedures and were waiting to be fed. The magnitude of the induced skin temperature shifts varied strongly, for instance, between 1.13°C (bat 1/V) and 5.75°C (bat 2/VI). Skin temperature shifts of similar size were also observed in other parts of the dark phases and even during some light phases. However, often they were not accompanied with a continuous calling activity. Therefore, we concentrated analyses only on activation periods, which were induced by the experimenter.

After the bats' activation, values of both skin temperature and *f*_rest_ increased to a maximum. Generally, frequency maxima (*f*_max_) were reached earlier than skin temperature maxima (*t*_max_). In the time span between *f*_max_ and *t*_max_, the skin temperature continued to increase and afterwards was maintained at the *t*_max_ level for some time before it was slowly reduced.

If it is indeed temperature that is determining *f*_rest_, the question remains as to why *f*_max_ was reached before the *t*_max_ of skin temperature. One explanation could be that the skin temperature, which we measured with our sensor between the shoulder blades, differed from the temperature in the cochlea, the location that is hypothesized to affect the adjustment of *f*_rest_. Several studies suggest that at constant ambient temperature, the skin temperature, measured in a neck fold or between the scapulae, gives a good approximation of body temperature (e.g. Audet and Thomas, 1996; Barclay et al., 1996; Henson et al., 1990; Huffman and Henson, 1993a; Willis and Brigham, 2003). However, during the warming-up phase of torpid bats, Willis and Brigham (2003) observed differences between the core temperature of body and skin temperature. Body temperature measured continuously with an implanted transmitter in the intraperitoneal cavity and compared with the skin temperature between the scapulae suggested that upon arousal from torpor, the increase rate in body temperature (0.52°C min^−1^) was higher than the increase rate in skin temperature (0.44°C min^−1^). This may explain why the body core temperature along with cochlear temperature reached their maxima earlier than skin temperature. Owing to the fact that the measured increase in skin temperature does not exactly reflect the increase of the core temperature and/or cochlea temperature, we cannot determine the average increase rate of frequency relative to body temperature.

At 10 of the 12 activations (except activations IV and VI of bat 2), a moderate increase in skin temperature led to the *f*_max_, which was reached between −2°C and the *t*_max_ and was maintained until the relative skin temperature had reached its maximum. Afterwards, the body temperature and *f*_rest_ slowly decreased, which was also accompanied by a decrease in skin temperature. The exceptions are activations IV and VI of bat 2, where the overall increase in skin temperature was higher. These two activations have in common that the skin temperature at beginning of the activation was distinctly lower, which indicates that the bat may have been in a state of deeper torpor in these situations, and which may explain these differing results.

From these experiments, we conclude that in *H. armiger*, body temperature determines CF_2_ of the emitted signals, and that an increase in temperature leads to an increase in emission frequency. According to the close phylogenetic relationship, we propose that this result may also hold for all hipposiderid and rhinolophid bats, although further testing of this hypothesis is warranted.

The influence of body temperature on the CF_2_ in a DS-compensating bat was only previously shown in the mormoopid *P. parnellii* (Huffman and Henson, 1993a). That study found that changes of body temperature correlated positively with the CF_2_ of the coupled *f*_rest_ and *f*_ref_ and also with the CRF. Further, flight and contralateral sound exposure also shifted CRF, and along with it the *f*_rest_ and *f*_ref_. Temperature also had influence on the offset between *f*_rest_ and *f*_ref_, which was reduced slightly with increasing temperature. The authors concluded that the induced changes of CRF were responsible for the observed changes of CF_2_ in stationary bats and in DS-compensating bats on a pendulum. They discussed the possible existence of a feedback mechanism for sound emission, where a ‘set point’ of neural activity in the vocal centres detects the coincidence of both the activation of the narrow band to which the cochlea is most sensitive, and the frequency of the emitted CF_2_. Huffman and Henson (1991, 1993b) complemented the temperature studies in *P. parnellii* by showing that the neuronal tuning of foveal neurons of the cochlear nucleus and of the inferior colliculus were also labile, and could change with temperature.

Further evidence that the tuning of the cochlea of DS-compensating bats depends on their physiological state comes from studies with anaesthetized bats. Audiograms of anaesthetized rhinolophids and hipposiderids differ conspicuously from those of non-anaesthetized bats, similar to observations of *P. parnellii* [Neuweiler, 1970: *Rhinolophus ferrumequinum*; Pollak et al., 1972: *Chilonycteris p. parnellii* (*P. parnellii*); Foeller and Kössl, 2000: *Hipposideros lankadiva*]. Further, in a study in which the physiological properties of the inner ear of the rhinolophid *Rhinolophus rouxii* and the mormoopid *P. parnellii* were directly compared, Henson et al. (1985) not only described the influence of anaesthesia on the sensitivity and tuning of audiograms in *R. rouxii*, but also suggested (without reporting corresponding data) that the cochlear microphonic audiograms were affected by temperature. Effects of temperature and anaesthesia on the foveal resonance system of *P*. *parnelli* were also reported from Kössl and Vater (1985), who measured cochlear microphonic potentials and evoked otoacoustic emissions.

All DS-compensating bats possess a mechanical resonance system, which is the basis for the auditory fovea, a highly expanded frequency representation in the range of CF_2_. Therefore, we propose similar influences on the foveal resonance system in the cochlea and concomitant shifts in CF_2_, regardless of the phylogenetic relationship of taxa. While acknowledging the marked differences in cochlear mechanics of the foveal tuning system in *P. parnellii* and in rhinolophid and hipposiderid bats (Henson et al., 1985), we assume that similarities in the effects of temperature and anaesthesia on the foveal tuning and the concomitant adjustment of CF_2_ as demonstrated in the present study for *H. armiger* and by others for *P. parnellii* indicate a similar audio–vocal control principle in all DS-compensating bats.

We found that the variation of body temperature of *H. armiger* results in a concomitant change in *f*_rest_. Owing to the coupling of *f*_rest_ and *f*_ref_, we are confident that temperature effects both frequencies in a similar way. The temperature-dependent variability of the CF_2_ allows conclusions on the function of the audio–vocal control system of DS-compensating bats. The controlled process variable of the audio–vocal feedback control system is the activation state of the foveal resonance area in the cochlea. This cochleo-topic activation status is reported to the audio–vocal control centre by the afferent foveal areas of the auditory pathway. The emission frequency is changed if the reported process variable differs from the set-point condition of the central control system. At deviations, some kind of push/pull mechanism changes via efferent motor pathways the emitted CF_2_, with inhibitory feedback lowering and excitatory feedback increasing the emitted CF_2_ (Metzner et al., 2002). A change of the emitted CF_2_ modifies, through feedback, the whole auditory input consisting of the emitted signal and all returning echoes until the foveal input into the vocal control centre has reached the set-point condition again. Flying bats perceive the auditory input consisting of the emitted signal and its delayed DS echoes and adjust the emission frequency in such a way that the highest echo frequency in the perceived pulse-echo train is, independent of flight speed, kept constant at the so-called reference frequency (average of echoes with highest DS returning from ahead) with a standard deviation of 0.1–0.2% (Schnitzler and Denzinger, 2011; Schoeppler et al., 2018). Resting bats perceive the auditory input consisting of the emitted resting signals and their delayed non-DS echoes and adjust the emission frequency at the so-called resting frequency (averaged emission frequency of stationary bats), which is kept constant within short periods (again with a standard deviation of 0.1–0.2%; Schnitzler and Denzinger, 2011; Schoeppler et al., 2018). The coupling between resting and reference frequency indicates that the activation state of the cochlea by the pulse-echo train of resting and of reference frequency is most likely similar if the two coupled frequencies are separated by the observed offset, between 50 and 300 Hz (Schnitzler and Denzinger, 2011). Our data from *H. armiger* and previous data from *P. parnellii* (Huffman and Henson, 1991, 1993b) suggest a similar mechanism of the audio–vocal control system in all other DS-compensating hipposiderids and rhinolophids.

We suggest here that the tuning of the hard-wired auditory fovea cannot be deliberately varied. According to the data collected, all reported changes of resting and/or reference frequency in DS-compensating bats originate from the audio–vocal control system either by morphological or physiological changes of the resonance system. Irreversible changes of CF_2_ in adult bats may be related to aging processes, which change the tuning properties of the foveal resonance system. The observation that wild living *R. ferrumequinum* aged 10–23 years (Jones and Ransome, 1993) dropped *f*_rest_ by approximately 200 Hz year^−1^ and that one individual of *H. armiger* and one individual of *Rhinolophus paradoxolophus* dropped CF_2_ by 1 and 0.9 kHz, respectively (D.S., personal observation) may be explained by age-related morphological changes in the inner ear. A well-studied example of how growth-related changes in morphology influence the CF_2_ is the increase of CF_2_ of young DS-compensating bats during ontogeny (for reviews, see Rübsamen, 1992; Vater et al., 2003).

We assume, that, if aging processes can be excluded, variations in *f*_rest_ and/or the coupled *f*_ref_ underlie reversible physiological mechanisms that influence the nature of the travelling wave. It has been shown that cochlear micromechanics changed with temperature. For instance, weak temperature effects were found in the motility of the outer hair cells of guinea pigs (reviewed in Ashmore, 2008). Though these effects seem to be marginal in other mammals, they may be distinctive in DS-compensating bats owing to the high expansion of the frequency representation at their auditory fovea.

For instance, changes of *f*_rest_ and/or *f*_ref_, which have been attributed to social interactions, may in fact be related to temperature effects (Hiryu et al., 2006; Furusawa et al., 2012). A strong support for our temperature hypothesis is the observation that a drop in body temperature was indicative of a subject that was close to death. Furusawa et al. (2012) described a large frequency drop of 2 kHz in a sick bat before it perished. Also, Hiryu et al. (2006) measured a significant decrease in *f*_rest_ before a bat died. We suggest that these sick bats reduced their body temperature and with it *f*_rest_. In future studies on the effects of social interactions on CF_2_, it should also be tested whether these interactions occur simultaneously with body temperature changes, which could also explain the observed frequency changes.

Besides DS-compensating bats, other vertebrates also have hearing systems that are influenced by body temperature. Changes in the sensitivity of audiograms and of also the tuning of single neurons have been reported for amphibians, reptiles and birds (e.g. Hubl et al., 1977; Walkowiak, 1980; Smolders and Klinke, 1984; Schermuly and Klinke, 1985). In guinea pigs, a correlation between the temperature and the characteristic frequency of neurons was not observed (Gummer and Klinke, 1983), but a loss in sharpness and an increase in threshold did occur. In the non-DS compensating bat *Myotis lucifugus*, temperature reduction led to a reversible decrease of sensitivity of the N1 response to all frequencies, with a greater effect observed at higher frequencies (Harrison, 1965).

In humans, a variation of the characteristic frequency of spontaneous otoacoustic emissions observed during menstrual or diurnal cycles, as well as during fever, has been discussed (Wit, 1985) and supported (Wilson, 1986; O'Brien, 1994) as an effect of body temperature. Bell (1992), however, found no body temperature effect, and suggested that more likely candidates were changes in hormonal or cardiovascular activity. Wynn (1972) found small variation in estimation of absolute pitch in both women and men, owing to hormonal cycle or illness. Strong evidence that hormones play a role in ear activity is the presence of beta1-adrenergic receptors in the organ of Corti, which Fauser et al. (2004) found in gerbils. They concluded that the sympatric innervation may enhance the potassium (K^+^) efflux in the inner and outer hair cells. Therefore, we cannot exclude that in DS-compensating bats, additional physiological factors may have an influence on the resonance properties of the cochlea.

### Conclusions

The observed increase of *f*_rest_ in warm-up phases of *H. armiger* reflect a change in the temperature-dependent controlled process variable of the audio–vocal control system, i.e. the cochleo-topic activation state of the foveal resonance area in the cochlea. A concomitant change in emission frequency occurs if the state that is reported to higher foveal centres deviates from the central set-point condition, owing to increases in body, and therefore cochlea, temperatures. We propose to generalize our conclusions to all other DS-compensating hipposiderids and rhinolophids bats, and also to the DS-compensating mormoopid bat *P. parnellii*, based on findings that this species reacts in a comparable way to changes in body temperature. The cochleo-topic organization of the feedback control system guarantees an undisturbed function of DS compensation independent of physiologically determined changes of emission frequency.

## Supplementary Material

Supplementary information
