# Supplementary material for: The resting frequency of echolocation signals changes with body temperature in the hipposiderid bat Hipposideros armiger
Source: J Exp Biol. 2022 Feb 3;225(3):jeb243569. doi: 10.1242/jeb.243569 (PMC8918815; doi:10.1242/jeb.243569)
Supplement: Supplementary information [file jexbio-225-243569-s1.pdf]

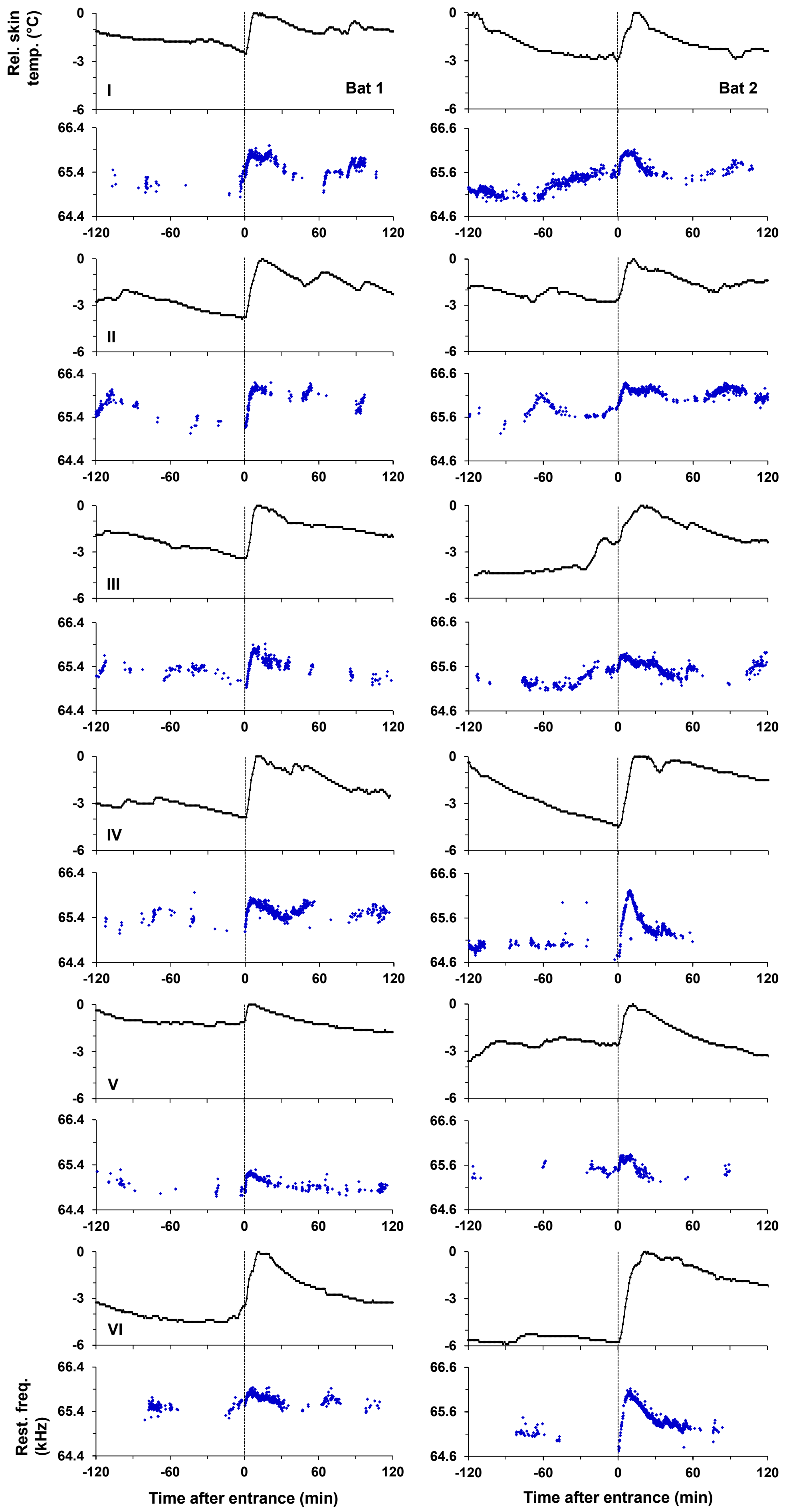

Journal of Experimental Biology • Supplementary information

Fig. S1. Relative skin temperature (Rel. skin temp.) and resting frequency (Rest. freq.) over a time course of two hours before and after activation by the experimenter. For each bat, six activations are shown**.** Skin temperature is depicted relative to the maximum temperature reached after each activation. The vertical lines mark the instant of time when the experimenter entered the husbandry room. Each frequency value represents the resting frequency in a five-second slot.
